# The Use of DREADDs to Deconstruct Behavior

**DOI:** 10.3389/fgene.2016.00070

**Published:** 2016-05-17

**Authors:** Paul D. Whissell, Sarasa Tohyama, Loren J. Martin

**Affiliations:** ^1^Department of Psychology, University of TorontoToronto, ON, Canada; ^2^Department of Psychology, University of Toronto MississaugaMississauga, ON, Canada

**Keywords:** chemogenetics, DREADD receptors, learning, depression, anxiety, pain, behavior, optogenetics

## Abstract

A central goal in understanding brain function is to link specific cell populations to behavioral outputs. In recent years, the selective targeting of specific neural circuits has been made possible with the development of new experimental approaches, including chemogenetics. This technique allows for the control of molecularly defined subsets of cells through engineered G protein-coupled receptors (GPCRs), which have the ability to activate or silence neuronal firing. Through chemogenetics, neural circuits are being linked to behavioral outputs at an unprecedented rate. Further, the coupling of chemogenetics with imaging techniques to monitor neural activity in freely moving animals now makes it possible to deconstruct the complex whole-brain networks that are fundamental to behavioral states. In this review, we highlight a specific chemogenetic application known as DREADDs (designer receptors exclusively activated by designer drugs). DREADDs are used ubiquitously to modulate GPCR activity *in vivo* and have been widely applied in the basic sciences, particularly in the field of behavioral neuroscience. Here, we focus on the impact and utility of DREADD technology in dissecting the neural circuitry of various behaviors including memory, cognition, reward, feeding, anxiety and pain. By using DREADDs to monitor the electrophysiological, biochemical, and behavioral outputs of specific neuronal types, researchers can better understand the links between brain activity and behavior. Additionally, DREADDs are useful in studying the pathogenesis of disease and may ultimately have therapeutic potential.

## Introduction

In recent years, the emergence of genetic techniques to selectively manipulate cellular activity has revolutionized the neurosciences. Two of the most commonly used genetic techniques to control cell activity are chemogenetics, which uses engineered G-protein coupled receptors that are activated by otherwise inert drug-like small molecules, and optogenetics, which uses channels that are activated by light (Boyden et al., 2005; Armbruster et al., 2007). The incorporation of chemogenetics and optogenetics into animal models has greatly advanced our understanding of neural circuits and redefined the drug development process. Beyond their use in exploratory research, these techniques also have considerable translational potential. Both chemogenetics and optogenetics have been applied in non-human primates and may ultimately have therapeutic utility in humans, particularly in the treatment of behavioral disorders (Urban and Roth, 2015).

Chemogenetic technology has been employed in many subfields of neuroscience, but is perhaps most frequently used to investigate the neural mechanisms of behavior. The accessibility and versatility of chemogenetic technology has resulted in an explosion of ‘end users’ and an increasing amount of behavioral labs now include the chemogenetic technique in their everyday ‘toolbox.’ Here, we summarize the practical considerations for the use of chemogenetics, and contrast this technique with optogenetics. Additionally, we review how chemogenetic approaches have been used to interrogate a wide range of behaviors, including associative learning, memory, cognition, feeding, mood, and pain. Finally, we discuss the use of chemogenetics in animal models of disease and comment on the future potential of chemogenetics in translational research and clinical settings.

### Practical Considerations

Chemogenetic technology allows for the precise characterization of specific cell types in the context of their native systems. To study a particular cell subpopulation using chemogenetics, engineered designer receptors must first be selectively expressed in that cell population. Chemogenetics in the context of this review refers to the designer receptors exclusively activated by designer drugs (DREADDs) developed by Bryan Roth and colleagues at the University of North Carolina (Armbruster et al., 2007). DREADDs are modified muscarinic G-protein coupled receptors (GPCRs). These designer receptors are typically introduced into cells by viral vectors and they provide a lock-and-key approach to selectively modulate cellular activity by chemical means.

DREADD technology has been used to control the activity of a wide range of cell types. The selective targeting of DREADDs to a cell population can be achieved by using a cell type-specific promoter to drive DREADD expression, and the expression of this promoter can be further controlled using a recombinase-based system. There are many viral constructs encoding DREADDs that require genetic recombination to produce functional designer receptors, most notably *Cre*-dependent adeno-associated viruses (AAVs). The use of *Cre*-dependent AAVs restricts DREADD expression to cells that selectively express *Cre*. Given the wide range of transgenic mouse lines in which *Cre* expression is restricted to a particular cell type, *Cre*-dependent AAVs are an increasingly popular method of selectively expressing DREADDs in specific cell populations. In cases where a viral construct is employed, spatial specificity of DREADD expression is further refined via stereotaxic microinjection of the virus into a particular location.

**FIGURE 1:**
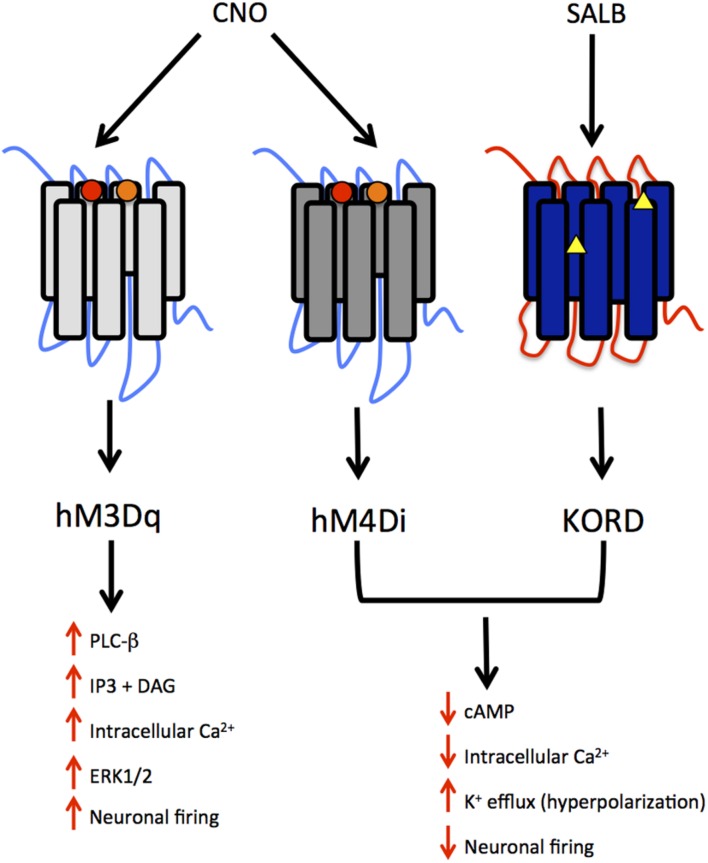
**Summary of modified human muscarinic (hM) and Kappa-opioid receptor (KOR) designer receptors exclusively activated by designer drugs (DREADD) subtypes. hM3Dq, human M3 - Gq coupling; hM4Di, human M4- Gi coupling; KORD – Gi coupling.** The muscarinic receptors are activated by clozapine N-oxide (CNO), a metabolite of clozapine and the KORD is activated by salvinorin B, an inactive, drug-like metabolite of the KOR-selective agonist salvinorin A (SALA). The intracellular signaling and neuronal activity that results from the activation of these receptors is also depicted. Circles represent mutations in TMDIII and TMV for muscarinic receptors whereas triangles represent mutations in the “message domain” of TMII and extracellular end of TMVI for KORD.

DREADDs possess a low affinity for endogenous ligands and little constitutive activity, but may be activated by synthetic compounds. Most DREADDs are selectively responsive to the orally available drug, clozapine N-oxide (CNO; Armbruster et al., 2007). Though CNO is a metabolite of the atypical antipsychotic medication clozapine, the compound is biologically inert in rodents and lacks appreciable affinity (*K*i > 1 μM) for all relevant native central nervous system targets (Armbruster et al., 2007). The effect of CNO on a DREADD depends upon the signaling cascade to which that DREADD is coupled. DREADDs, much like endogenous GPCRs, are either coupled to an inhibitory (Gi) or excitatory (Gq, Gs) signaling cascade. CNO activation of the modified human M4 muscarinic DREADD, which is coupled to G_i_ signaling, silences neuronal activity (hM4Di receptor; Armbruster et al., 2007). Conversely, CNO activation of the modified human M3 muscarinic DREADD receptor, which is coupled to Gq signaling, elicits burst firing in neurons (Alexander et al., 2009; hM3Dq receptor; see **Figure 1**).

The temporal properties of DREADD activation are dependent upon the pharmacokinetic properties of their agonists. Following peripheral administration of CNO, plasma levels of the drug peak within 30 min and sharply decline over the subsequent 2 h (Guettier et al., 2009). Though plasma levels of CNO decline quickly, behavioral effects of the drug may be evident for up to 6 h (Alexander et al., 2009). Thus, DREADD technology is preferred in studies where the activity of neurons must be manipulated over a longer time period (hours to days). In contrast, a technique such as optogenetics is strongly preferred for studies where neuronal activity must be controlled over shorter time intervals (seconds to milliseconds).

DREADD approaches are highly flexible in terms of drug delivery. CNO is commonly administered via injection, but the drug can also be mixed into food chow or drinking water (Urban et al., 2016). The delivery of CNO via food or water may be convenient in long-term studies, particularly if the experimenter wishes to avoid repeated handling and/or injecting of animals. Though food and water are less invasive routes of administration for CNO, they are also less precise. It is difficult to control for the amount of CNO ingested via these routes, as the intake of food and water may fluctuate. An alternative method of delivering CNO, which has yet to be widely used, is the implanted osmotic minipump. This device could release a well-controlled systemic dose of CNO over a long period of time.

**FIGURE 2:**
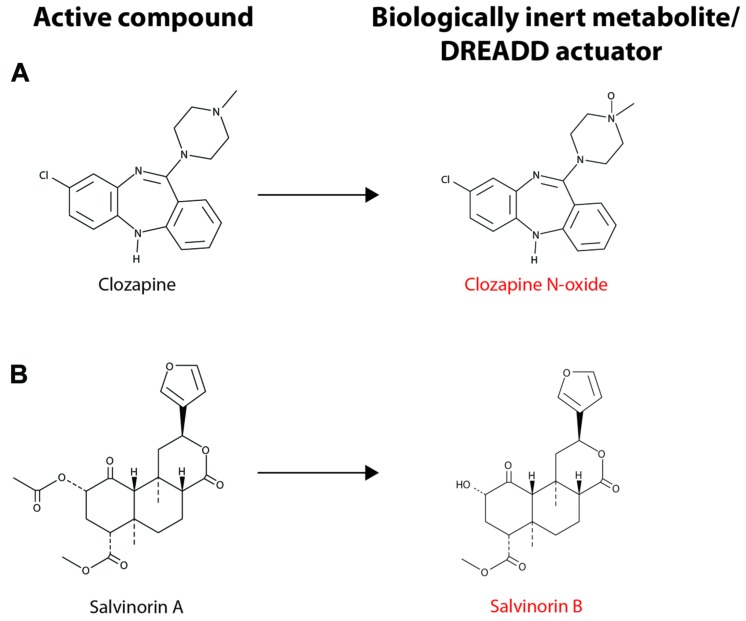
**Chemical structure of DREADD actuators. (A)** The chemical structures for clozapine and clozapine N-oxide (CNO) are shown. **(B)** The chemical structures for salvinorin A (SALA) and salvinorin B (SALB) are shown. CNO and SALB are otherwise biologically inert and are considered selective DREADD actuators. CNO stimulation of hM3Dq- or hM4Di-expressing neurons leads to neuronal excitation or inhibition, respectively (see text for details). Activation of KORD by SALB leads to neuronal inhibition, albeit on a quicker time scale than the activation of hM4Di by CNO. Note that CNO differs from clozapine only in the presence of the N-oxide moiety and SALA undergoes deacetylation to form SALB.

Though CNO-dependent DREADDs have proven very useful in rodent models, there is a strong interest in developing new DREADDs with alternative properties for several reasons. Although CNO is not subject to significant metabolic transformation in mice and rats, a small fraction of the drug is reverse metabolized to clozapine in humans, non-human primates and guinea pigs (Jann et al., 1994; Chang et al., 1998; Loﬄer et al., 2012). The conversion of CNO to clozapine in several species limits the scope and translational potential of CNO-dependent DREADDs. Recently, a novel DREADD has been developed that incorporates an engineered kappa-opioid receptor coupled to an inhibitory G_i_-signaling cascade. This kappa-opioid receptor DREADD (termed KORD) is insensitive to endogenous ligands, including the dynorphins, which are the natural ligands for kappa-opioid receptors. The KORD is exclusively activated by the inert ligand, salvinorin B (SALB), which is a semi-synthetic analog of the natural psychotropic agent salvinorin A (Vardy et al., 2015). A comparison of the chemical structures of DREADD agonists, and the compounds from which these agonists are derived, is presented in **Figure 2**. The development of more sophisticated DREADDs that offer better control and specificity will increase the translational potential of DREADD technology.

**FIGURE 3:**
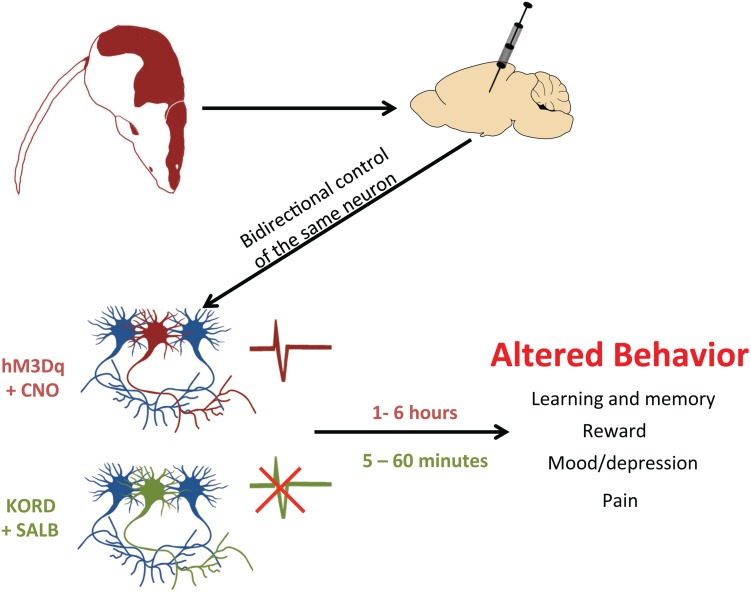
**Using DREADDs to deconstruct behavior.** In order to determine whether neurons drive a particular behavior, a DREADD can be expressed by microinjection into restricted cell populations. With two different DREADDs, responding to different exogenous ligands, the bidirectional control of neuronal activity is possible. By activating the excitatory DREADD (hM3Dq, red neuron) via CNO, it is possible to see which cell populations control certain behaviors. Alternatively, this same set of neurons can also express the inhibitory KORD (green neuron), which when activated by SALB will silence neuronal firing. Since CNO-activated DREADDs operate on a long timescale (1–6 h) and KORD-activated DREADDs have a rapid time course (5–60 min), experimenters can now excite a particular set of neurons and then rapidly inhibit them to provide conclusive evidence of their involvement in a given behavior.

The existence of multiple DREADDs with different ligand affinities also allows for another benefit: the incorporation of multiple DREADDs into the same biological system (Vardy et al., 2015; see **Figure 3**). In a recent study, excitatory (hM3Dq) and inhibitory (KORD) DREADDs were expressed within the same animal (Vardy et al., 2015). This ‘multiplexed’ approach permitted the bidirectional control of neuronal activity and a more thorough interrogation of behavior. Importantly, as the pharmacokinetic profiles of SALB and CNO differ – SALB mediates rapid short-lasting effects (≈5 min) whereas CNO mediates delayed long-lasting effects (≈1 h) – each drug differentially affects the temporal dynamics of neuronal activity (Vardy et al., 2015). Thus, CNO-based DREADDs and SALB-based DREADDs may each provide unique information about neuronal function. SALB-based DREADDs may be more informative of how transient inactivation of neurons affects behavior whereas CNO-based DREADDs may be informative of how tonic activation of neurons influences behavior. Users of DREADD technology should be aware that acute and tonic activation of neurons are not equivalent and may have different – even opposite – behavioral consequences. This was illustrated in a recent study, wherein acute and chronic activation of serotonergic neurons had different effects on mood (Urban et al., 2016).

### DREADDs and Optogenetics

In order to fully appreciate the utility of DREADD technology, it is necessary to understand how this technique differs from other approaches, most notably optogenetics. Here, we briefly review some of the major differences between DREADDs and optogenetics that have implications for the study of behavior. For more detailed comparisons of these two techniques, the reader is directed to several excellent reviews (Aston-Jones and Deisseroth, 2013; Krashes and Kravitz, 2014; Krook-Magnuson and Soltesz, 2015; McElligott, 2015; Lee and Kim, 2016).

A primary difference between DREADDs and optogenetics is the degree of temporal specificity in controlling neuronal activity. As mentioned previously, DREADDs are ideally suited for prolonged modulation of cell activity in the range of minutes–hours (Armbruster et al., 2007; Alexander et al., 2009). Optogenetics, in contrast, can reversibly manipulate cell activity in the range of milliseconds. With optogenetics, the experimenter has direct control over the frequency of light stimulation and therefore the frequency of neuronal activity. Many studies utilize ‘physiologically relevant’ patterns of light stimulation that model endogenous patterns of neuronal activity (Kim et al., 2016). The difference in temporal resolution between DREADDs and optogenetics is critical when deciding which technique is best for an experiment. If a researcher is interested in modulating neuronal activity during a brief behavioral state, such as an episode of freezing behavior, optogenetics may be most suitable. Alternatively, DREADDs might be more ideal if the researcher wishes to modify neuronal activity over a prolonged time period, such as during a 30 min period of restraint stress.

DREADDs and optogenetics both have a relatively high degree of spatial resolution, as both can be made cell-specific. In optogenetic studies, researchers achieve an especially high level of spatial resolution through controlled placement of the optic fiber. In the case of DREADDs, a comparable level of spatial specificity is only possible if there is restricted delivery of the exogenous ligand to a particular brain area, such as via intracranial microinjection. This approach can be effective in dissecting specific pre- or post-synaptic mechanisms (Vazey and Aston-Jones, 2014). An additional consideration for the use of optogenetics is that the effects of the applied light may not be restricted to the axons or terminals of interest. Indeed, excitation of terminals with light may lead to backpropagation of excitatory signals to the cell body (Grossman et al., 2013).

DREADDs offer many of the same advantages as optogenetics, but are arguably easier to implement. As mentioned above, the experimenter has considerable flexibility in a DREADD study, and may administer CNO via several routes (injection, food, water, or pump). In contrast, optogenetic studies require specialized instrumentation to administer light, including waveform generators, optic fibers and other devices (Zhang et al., 2010). In optogenetic studies, the animal often has a tethered implant, which may make measurement of certain behaviors difficult.

Both DREADDs and optogenetics have inherent advantages and disadvantages. The technique that is most useful depends upon the experimental question and the resources available. These techniques should be considered complimentary, not competitive, and can even be employed within the same experimental design. It is important to note that most studies have found few significant differences comparing DREADD and optogenetic approaches (Krashes et al., 2011; Carter et al., 2013; Zhan et al., 2013; Siuda et al., 2016); however, there are some exceptions to this (Carreno et al., 2015).

## Interrogation of Behavior Using DREADDs

Neuroscientists routinely express DREADDs in specific brain regions, usually via viral microinjections. Importantly, DREADD expression in the brain is also possible without viral injection or invasive surgery. Recently, transgenic mice have been generated that constitutively express DREADDs in specific cellular populations. Notably, a mouse model has been developed that exclusively expresses hM3Dq in glial fibrillary acidic protein^+^ (GFAP^+^) glial cells (Agulhon et al., 2013), which are non-neuronal cells that play an important support function in the nervous system. The activation of hM3Dq in GFAP^+^ glial cells increased heart rate and blood pressure in a manner that was not dependent upon intracellular Ca^2+^ release. Others have shown that stimulating glial cells by activating hM3Dq receptors with CNO generated neurogenic contractions in the ileum and colon, suggesting that enteric glial cells may play a critical role in controlling gut reflexes (McClain et al., 2015). Another study has shown that selectively activating glial cells using DREADDs hinders cocaine-seeking behavior in rats (Scofield et al., 2015). DREADDs have also been useful in investigating other non-neuronal cells besides glia, such as cardiac cells (Kaiser et al., 2015), breast cancer cells (Yagi et al., 2011), pancreatic β-cells, and hepatocytes (Hua Li et al., 2013). Although DREADDs have been used to study non-neuronal cells, the current review will focus exclusively on the use of DREADDs in neurons.

A novel DREADD-based method for studying neural circuits in freely moving animals is the “DREAMM” (DREADD-assisted metabolic mapping) technique (Michaelides et al., 2013; Urban et al., 2016). This method combines DREADD technology with behavioral μPET imaging to generate whole-brain metabolic maps. The initial study describing the utility of DREAMM used herpes simplex virus (HSV) vectors to express hM4Di receptors into specific populations of medium spiny neurons (MSN) in the nucleus accumbens. Next, in order to determine the whole brain consequence of MSN inhibition, rats were scanned using μPET following CNO administration. The inhibition of MSNs was highly associated with increased activity in the primary components of the limbic system including the ventral palladium, amygdala, entorhinal cortices, and hippocampus. In contrast, MSN inhibition also led to significant decreases in activity in the ipsilateral sensory cortex, globus pallidus, and contralateral piriform cortex. Overall, these findings identified distinct neural activity associated with MSN inhibition and emphasized that impairments in the nucleus accumbens pathway have profound bottom-up effects on cortical activity, particularly in the limbic system (Michaelides et al., 2013). DREAMM has the potential to quantitatively delineate discrete changes in whole-brain neuronal circuits *in vivo* and assess cell-type–specific whole-brain neuronal circuits during the awake state. Overall, DREAMM fills a technological niche, but can also be applied to many areas of neuroscience to advance our understanding of whole-brain neural networks and functional connectivity.

Recently, chemogenetic technology has been extended from rodents to monkeys. In one remarkable study, hM4Di receptors were used to disrupt the connections between the rhinal and orbitofrontal cortices (OFC) (Eldridge et al., 2016). The disruption of this pathway resulted in diminished sensitivity to differences in reward value. These results are an important extension of previous findings (Clark et al., 2013), and illustrate the translational potential of DREADD technology.

With the recent surge in studies using DREADD techniques, there exists a plethora of papers that provide further insight into the neural mechanisms of various behaviors (Ferguson and Neumaier, 2012; Lee et al., 2014; Urban and Roth, 2015; Roth, 2016; Smith et al., 2016). An excellent review was recently published that highlights DREADD applications in behavioral neuroscience (Smith et al., 2016). In their review, Smith et al. (2016) briefly highlight the use of DREADDs to study learning, memory and drug addiction with a particular emphasis on strategies that allocate specific neurons to these behaviors. Herein, we expand upon this and highlight key studies that use DREADDs to deconstruct a broad range of behaviors including learning, memory, mood, feeding, and pain. Based on these findings, we extrapolate the therapeutic value of DREADDs for drug discovery and treating various disease states.

### Associative Learning

Understanding the mechanisms of learning is a longstanding goal of neuroscience, and this pursuit has been greatly facilitated by DREADD techniques. Several recent studies have used DREADDs to investigate associative learning (Robinson et al., 2014; Yau and McNally, 2015), a process thought to be involved in behavioral tasks such as sensory preconditioning and fear conditioning.

Sensory preconditioning is a type of learning that requires forming stimulus-stimulus associations (Robinson et al., 2014). While it is widely accepted that preconditioning involves the hippocampus (Yu et al., 2014), it is unclear which other regions participate. Robinson et al. (2014) investigated whether the retrosplenial cortex (RSC), a structure interconnected with the hippocampus, is involved. In their model, hM4Di receptors were selectively expressed in the neurons of the RSC. First, animals were trained on a sensory preconditioning trial, wherein a light and tone stimulus were presented together (light-tone pairing). Thereafter, during a conditioning trial, the light stimulus was presented with food (light-food pairing). Animals that acquired the light-food association demonstrated a conditioned food-seeking response to light. Further, animals that also acquired the light-tone association during the sensory preconditioning trial further showed a conditioned food-seeking response to the tone stimulus – even though the tone had never been paired with food. It was found that injection of CNO during the preconditioning trial, which inhibited hM4Di-RSC neurons, reduced the conditioned food-seeking response to the tone. Accordingly, the authors concluded that sensory preconditioning requires activity in the RSC.

New associative learning is thought to occur whenever a predicted outcome for an event differs from the actual outcome – a ‘prediction error’ (Schultz and Dickinson, 2000). Animals, much like humans, appear to best recall their ‘mistakes,’ and demonstrate learning following errors in predicting outcomes. The neural circuitry of prediction error has been studied using DREADDs by Yau and McNally (2015), who examined the contribution of the dorsal medial prefrontal cortex (dmPFC) by expressing hM3Dq receptors in pyramidal neurons within this region (Yau and McNally, 2015). First, hM3Dq-dmPFC mice underwent a fear conditioning trial wherein a tone (CS1) was paired with a footshock (CS1-shock pairing). As a result, mice exhibited strong conditioned fear to CS1. Subsequently, the mice were re-trained on the task, only the footshock was now paired with the simultaneous presentation of the tone (CS1) and a light (CS2) stimulus (CS1 + CS2-shock pairing). While animals maintained a conditioned fear response to CS1 after this training protocol, they did not acquire a conditioned fear response to CS2. This ‘blocking’ of conditioning to CS2 is thought to occur because of the lack of prediction error – CS1 was already a strong predictor of an impending foot shock, thus the CS1 + CS2 combination generated little prediction error for CS2. Interestingly, the activation of hM3Dq-dmPFC neurons with CNO during the combined stimulus trial (CS1 + CS2) prevented the blocking of fear conditioning to CS2. This result implies that the excitation of dmPFC neurons can facilitate learning new associations, perhaps by altering prediction error during conditioning trials.

### Memory

As well as studying the mechanisms of associative learning, DREADD approaches have also been applied to dissect the neural substrates of memory. In the brain, acquired memories are represented by the collective activity of discrete ensembles of neurons, referred to as memory traces (Tonegawa et al., 2015). Of late, the physiological factors governing the encoding, consolidation and updating of memory traces have been extensively studied using DREADD approaches.

In particular, DREADDs have afforded considerable insight into how memory traces are encoded (Sano et al., 2014; Yiu et al., 2014). Most traces involve only a fraction of neurons in a given brain region (~10–30% of lateral amygdala neurons may represent a fear memory, for example) and the process by which neurons are included is thought to be competitive. Using DREADD and other techniques, Yiu et al. (2014) addressed whether the excitability of neurons during encoding affected their probability of being incorporated into a memory trace. In testing this possibility, Yiu et al. (2014) selectively expressed hM3Dq receptors in lateral amygdala neurons and injected mice with CNO prior to training in a fear conditioning task. If excitability were a factor in determining the composition of the memory trace, then CNO treatment during training should increase the likelihood that hM3Dq+ neurons are incorporated into the trace. Indeed, the authors observed this finding: CNO injection before training greatly increased the probability that hM3Dq+ neurons were active during retrieval of the fear memory, suggesting that these neurons represented the fear memory trace. To further verify this interpretation, the authors tested whether reactivation of these hM3Dq+ neurons resulted in the expression of fear. In hM3Dq+ animals given CNO during training, the injection of CNO led to fear expression even in absence of the conditioned stimuli normally required to elicit this response. These findings strongly suggest that the neurons that are most excitable during fear conditioning are incorporated into the fear memory trace. Interestingly, another lab used DREADD technology to demonstrate that similar principles govern the assembly of memory traces for conditioned taste aversion in the insular cortex (Sano et al., 2014).

The prolonged process of memory consolidation follows memory encoding. Recently, the timeline for the consolidation of contextual fear memory was investigated using DREADD techniques (Zhu et al., 2014). In this study, hM4Di receptors were selectively expressed in CAMKIIα neurons in the hippocampus. Mice underwent training in the fear conditioning task and were subsequently given an injection of CNO at either 0–4 h or 6–10 h after training. Strikingly, mice given an injection 0–4 h after training but not 6–10 h after training demonstrated impaired contextual fear conditioning. Surprisingly, the authors found that the inhibition of CAMKIIα neurons in the ventral hippocampus (vHPC) impaired the consolidation of contextual fear memory – inhibiting neurons in the dorsal hippocampus had no effect. These results suggest that the ‘window’ for memory consolidation of contextual fear memory in the vHPC is within 6 h of training. Similar rules appear to govern the consolidation of other memory traces, including those associated with drugs of abuse such as cocaine (Shi et al., 2015).

DREADD techniques have also been applied to study the time-dependent reorganization of memory (Richards et al., 2014). Over time, related memories are thought to collectively form ‘schemas’ which are defined by the concepts that these memories share, and this process likely involves the mPFC (Brod et al., 2015). Richards et al. (2014) tested this theory using an innovative approach. In the water maze task, mice were trained to navigate toward several platform locations over a series of trials, with all locations being clustered in one region of the maze. This paradigm was thought to encourage the development of a spatial schema that would allow mice to reliably predict the location of the platform. At 1 and 30 days after this protocol, the ability of mice to acquire novel platform locations consistent or inconsistent with this schema was assessed. Congruent with the idea that schemas develop over time, mice tested 30 days after training more efficiently navigated toward a schema-consistent platform. Interestingly, mice tested 30 days after training also demonstrated better recall of a schema-inconsistent platform location during a probe trial. The authors speculated that mice tested 30 days after training exhibited stronger recall of the schema-inconsistent platform because of high prediction error during acquisition of its location. To test whether the mPFC was important for the enhanced recall of the schema-inconsistent platform location, the authors used DREADD techniques. In mice with hM4Di receptors selectively expressed in PFC neurons, the application of CNO during the acquisition of a schema-inconsistent platform location reduced subsequent recall of this location in a probe trial. These results suggest that the mPFC is important to spatial schemas and also support the notion that the mPFC contributes to learning as a result of prediction error [as suggested by Yau and McNally (2015)].

### Reward-Guided Behaviors

DREADDs have also been applied to study more general processes, including reward-guided behaviors (Chang et al., 2015; Ward et al., 2015). Typically, animals will form a strong association between a reward and any cue that is present at the time the reward is delivered (reward-cue pairing). Subsequently, the presentation of a reward-paired cue will elicit a reward-seeking response toward that cue. For example, if a lever (cue) is extended whenever food (reward) is presented, an animal will approach and interact with the lever whenever it is presented (reward-seeking behavior). This behavior, termed sign tracking, occurs even though the lever itself is not rewarding and manipulating the lever is not required for the reward to be obtained. Sign tracking is thought to occur because the incentive value of the reward has been transferred to the cue, but its mechanisms are poorly understood. Using DREADD techniques, Chang et al. (2015) demonstrated that sign tracking importantly involves the ventral pallidium (VP) of the brain. In their experiment, a lever (CS+) was presented in conjunction with a food reward. As a result, animals demonstrated a sign-tracking response (lever pressing) whenever the lever is extended. To directly manipulate VP activity, neurons of the VP were transfected with hM4Di receptors. Thirty minutes prior to the acquisition period, when animals learn to pair the CS+ with the reward, CNO was injected to inactivate the VP. Interestingly, CNO injection impaired the ability to acquire lever pressing in response to the CS+. These results demonstrated an important role of VP neurons in the acquisition of sign-tracking.

Evaluating the reward probability of an action is an important factor in decision-making, and the OFC is thought to play a central role in this process (Ward et al., 2015; Eldridge et al., 2016). Ward et al. (2015) studied the role of the OFC in this process using DREADD techniques. The authors generated mice in which hM4Di receptors were expressed on OFC neurons so that the activity of the OFC could be transiently inhibited. In this study, animals were taught that food rewards could be obtained via lever-pressing in two situations: high probability of reward and low probability of reward. Normally, animals were more accurate in obtaining food rewards in the high probability condition than in the low probability condition. This finding suggested that animals could discriminate between the reward probabilities and tailored their responses accordingly. When CNO was injected during testing, this discrimination between high and low reward conditions was not evident, and similar accuracy of responses was observed in both conditions. Interestingly, involvement of the OFC in reward-guided behavior has also been demonstrated using DREADD techniques in non-human primates (Eldridge et al., 2016).

Although reward-mediated behaviors have been extensively studied using DREADDs, this field of research should be complimented with a technique where precise temporal control is achievable, such as optogenetics. Decision-making operates on a millisecond timescale, and the ability to rapidly modulate neuronal activity with optogenetics may offer more insight into this process. Optogenetic manipulation of neuronal activity may change the decision-making process such that spilt-second decisions and behavioral outcomes are rapidly and bidirectionally altered.

### Feeding and Energy Expenditure

Given the immense heterogeneity that exists in the brain, it is absolutely essential to have tools that selectively target specific cell types. The neurons of the hypothalamus are diverse and control a large variety of behaviors, including feeding. With the development of techniques such as DREADDs and optogenetics, research into the neural circuitry of feeding behavior has made impressive progress.

Bradford Lowell, along with others including Michael Krashes, Scott Sternson, and David Olsen, have been at the forefront of using DREADD and optogenetic approaches to dissect feeding circuits (Krashes et al., 2011, 2013; Atasoy et al., 2012; Kong et al., 2012). Their research has largely focused upon how feeding behavior is regulated by agouti gene-related peptide (AgRP)-expressing neurons in the arcuate nucleus (ARC) of the hypothalamus. DREADD-mediated stimulation of ARC AgRP-neurons using hM3Dq receptors increases food intake, resulting in weight gain, less energy expenditure and enhanced fat storage (Krashes et al., 2011, 2013, 2014; Atasoy et al., 2012; Denis et al., 2015). A single injection of CNO (0.3 mg/kg) is sufficient to enhance feeding behaviors over several hours. Additionally, activation of AgRP neurons enhances feeding in calorically replete mice that have otherwise no motivation to consume food (Krashes et al., 2011).

With a sophisticated combination of mouse genetics and DREADDs, Krashes et al. (2013) were able to demonstrate that GABA and/or neuropeptide Y (NPY) is required for the rapid stimulation of feeding. Further, the authors showed that AgRP, through action on melanocortin 4 receptors (MC4Rs), is sufficient to induce feeding over a delayed yet prolonged period. Krashes et al. (2013) used a *Cre-*dependent AAV to express hM3Dq receptors in the AgRP neurons of triple knockout mice that lacked GABA release in these cells, as well as the genes for NPY and MC4Rs (*Agrp-Ires-Cre; Mc4r^-/-^; Vgat^flox/flox^; Npy^-/-^)*. This study has helped to elucidate the neurochemical mechanisms of AgRP neurons in controlling the distinct phases of eating.

These reports, though highly informative, represent only the “tip of the iceberg” in terms of DREADD-based research into feeding and energy expenditure. In addition to AgRP neurons, neurons expressing rat insulin promoter (RIP) have been linked to energy expenditure. The disruption of GABAergic synaptic transmission in RIP-*Cre* neurons of the ARC has been shown to reduce energy expenditure and increase obesity in mice (Kong et al., 2012). Additionally, mice with disrupted transmission were extremely sensitive to high fat diet-induced obesity, most likely due to defective diet-induced thermogenesis. When RIP-expressing neurons were activated using hM3Dq receptors, energy expenditure significantly increased but food consumption was unaffected (Kong et al., 2012). It was concluded that GABA plays an important role in regulating energy expenditure, but not feeding behavior.

Lastly, activation of pro-opiomelanocortin (POMC)-expressing neurons in the ARC and the nucleus tractus solitarius (NTS) using hM3Dq receptors has been shown to inhibit feeding behavior (Zhan et al., 2013). Whereas NTS POMC neurons induced appetite suppression via acute activation, ARC POMC neurons required prolonged activation. These results suggest that POMC neurons in the brainstem and hypothalamus regulate satiety differently. Through the use of transgenic mouse models and molecular tools that can monitor neural activity in freely moving mice, the ambitious goal of deconstructing the complex neural networks responsible for feeding behavior is fast becoming a reality.

### Mood

DREADD techniques are increasingly being applied to study mood disorders. Disorders of anxiety and depression are among the most prevalent mental health conditions, and carry with them a significant burden of disease (World Health Organization [WHO], 2008). A continual barrier in the treatment of these chronic and debilitating disorders is that we do not understand their underlying neural mechanisms. In determining the neurological bases of mood disorders, DREADD techniques have been a major asset.

One of the greatest challenges in treating mood disorders has been understanding the complex role that is played by the neurotransmitter 5-hydroxytryptamine (serotonin). The serotonergic system, which includes the transmitter as well as its receptors and transporters, plays a pivotal role in mood regulation and is often disrupted in mood disorders. In the treatment of anxiety disorders and severe cases of depression, selective serotonin reuptake inhibitors (such as fluoxetine) are commonly prescribed. The precise mechanism by which these drugs are therapeutic remains the subject of much debate, but is thought to involve both acute and chronic changes in serotonergic activity.

In understanding the contribution of the serotonergic system to mood, DREADD techniques have proven useful (Teissier et al., 2015; Urban et al., 2016). In one recent study, the effect of bidirectionally modulating the activity of serotonergic neurons in the raphe nuclei was investigated (Teissier et al., 2015). This study utilized *Pet1-Cre* mice, in which the expression of *Cre* is restricted to *Pet1*-expressing neurons, which are primarily serotonergic neurons of the mid-hindbrain. These *Pet1-Cre* mice were subsequently crossed with one of two Cre-responsive DREADD mouse lines (RC:PDi or RC:PDq) to generate transgenic mice that expressed either hM3Dq receptors (Pet1-Cre; RC:PDq mice) or hM4Di receptors (Pet1-Cre; RC:PDi mice) in raphe serotonergic neurons. Interestingly, the authors found that CNO injection in otherwise healthy hM3Dq mice increased anxiety-like behavior in the open field and elevated plus task maze tasks. Conversely, the injection of CNO in hM4Di mice had no effect. These results suggest that acute enhancement of serotonergic activity, but not inhibition, is anxiogenic.

Additionally, the authors investigated whether the effects of acutely manipulating raphe serotonergic activity were affected by changes in neurophysiology (Teissier et al., 2015). Notably, depressed patients exhibit substantially different neurophysiology relative to non-depressed individuals (Kohler et al., 2016), and may respond differently to manipulation of raphe serotonergic activity. To test the possibility that the effects of raphe serotonergic activity may change in depression, the authors conducted a similar series of experiments in a mouse model of depressive-like behavior (Teissier et al., 2015). In this model, mice were given fluoxetine during the perinatal period (from postnatal days 2 to 11) before being subjected to behavioral experiments in adulthood. These fluoxetine-treated animals, termed PNFLX mice, exhibit a depressive-like phenotype. Surprisingly, CNO treatment in PNFLX mice with expression of hM4Di receptors in serotonergic neurons reduced depressive-like behavior. This effect contrasts with the effect of serotonergic neuron inhibition in healthy control mice, which is minimal. The authors speculated that different effects of manipulating serotonergic neurons between healthy mice and PNFLX mice may be explained by comparative differences in the balance of serotonergic activity. In healthy mice, stimulation via hM3Dq activation may disrupt normal serotonergic activity and impede behavior. In contrast, in depressed mice, inhibition via hM4Di activation may normalize hyperserotonergic activity and rescue behavior.

Additionally, the temporal dynamics of serotonergic activity were shown to be an important factor in mood regulation, as short- and long-term activation of serotonergic neurons had contrasting behavioral effects (Urban et al., 2016). In this study, the expression of hM3Dq receptors was restricted to adult serotonergic neurons in the dorsal raphe nucleus (DRN). Short-term activation of DRN neurons, via CNO injection 30 min before behavioral testing, increased anxiety-like behavior and marginally reduced depressive-like behavior. Conversely, long-term activation of the serotonergic system, via the administration of CNO in the drinking water for 3 weeks prior to behavioral testing, reduced depressive-like behavior without affecting anxiety. These findings illustrate the stark differences between short- and long-term modulation of serotonergic activity. Additionally, these results shed light on the paradoxical increase in anxiety that is sometimes observed after commencing antidepressant treatment (the so-called ‘jitteriness syndrome’), which often dissipates followed continued treatment. Additionally, the finding that long-term CNO treatment is required to reduce depressive-like behavior is consistent with the observed treatment course of antidepressants, which often take weeks to show positive effects.

DREADD techniques have also been applied to study the mechanisms by which drugs exert their antidepressant effects (Carreno et al., 2015). The anesthetic drug ketamine, an *N*-methyl-D-aspartate antagonist, has recently attracted considerable attention for its rapid antidepressant effects. Currently, the mechanism of these effects is unknown. In a recent study, Carreno et al. (2015) demonstrated that afferent connections from the vHPC to the mPFC were critical for the long-term antidepressant effects of ketamine. In the forced swim test, mice given ketamine show less immobility and more swimming and climbing relative to controls, a result indicative of decreased depressive-like behavior. The long-term antidepressant effects of ketamine were abolished when the connections from the vHPC to the mPFC were blocked via lidocaine infusion. Using DREADD techniques, the authors showed that the selective activation of vHPC terminals in the mPFC had antidepressant actions strikingly similar to the effects of ketamine. In mice expressing hM3Dq receptors selectively on vHPC terminals in the mPFC, CNO injection reduced immobility and increased swimming and climbing in the forced swim test. Interestingly, the authors obtained an entirely different result when using an optogenetic approach to activate these vHPC-mPFC connections. Optogenetic stimulation of channelrhopdosin2-expressing vHPC terminals in the mPFC failed to enhance immobility, and did not appear to have an antidepressant effect. These results not only highlight the usefulness of DREADD techniques in dissecting neural circuitry of mood, but also are an important reminder that optogenetic and DREADD approaches may sometimes differ.

Other DREADD studies have revealed an important contribution of inhibitory circuitry in the mPFC to mood regulation (Perova et al., 2015). The inhibitory neurotransmitter γ-aminobutyric acid (GABA) is thought to be centrally involved in mood regulation and disruptions of the GABAergic system are often observed in mood disorders (Luscher et al., 2011; Whissell et al., 2014). Recently, several studies have utilized DREADD techniques to examine the contribution of GABAergic interneurons to mood. Perova et al. (2015) used DREADD techniques to examine the role of parvalbumin (PV)-expressing GABA neurons in the development of learned helplessness, a model of depression. In animals showing learned helplessness, PV-GABA neurons in the mPFC receive less excitatory input, a finding that suggests that reduced PV-GABA neuron activity may facilitate learned helplessness. To test this hypothesis, the authors selectively expressed hM4Di receptors in PV-GABA neurons of the mPFC in order to inactivate these cells. Indeed, Perova et al. (2015) observed that CNO treatment in hM4Di-PV-GABA mice promoted learned helplessness, verifying that reductions in PV-GABA neuron activity are associated with dysregulated mood.

In a further study, the contribution of somatostatin (SST)-expressing GABA neurons in the mPFC to emotional behavior was investigated (Soumier and Sibille, 2014). As GABAergic transmission tends to be reduced in mood disorders, the authors were specifically interested in the effects of inhibiting SST-GABA neuron function. To this end, they selectively expressed hM4Di receptors in SST-GABA neurons in the mPFC (Soumier and Sibille, 2014). As mood disorders involve both acute and prolonged disruptions of GABAergic activity, the effects of both short-term and long-term inhibition of SST-GABAergic function on emotionality were investigated. Emotional behavior was examined using a combination of assays, including the elevated plus maze, open field, novelty-suppressed feeding, sucrose preference and cookie tests. When SST-GABA neurons were acutely inhibited via CNO injection 30 min prior to behavioral testing, anxiety-like behavior and overall emotionality was increased. The effect of long-term inhibition of SST-GABA neurons was strikingly different. In the long-term treatment regime, CNO was injected twice per day over a period of 3 weeks. Surprisingly, chronic inhibition of SST-GABA neuron function decreased overall anxiety-like behavior and emotionality. These results highlight the value of DREADD techniques, which are ideally suited to study behavioral changes over a long time window.

Several studies have also employed DREADD techniques to examine the contribution of dopamine (DA)-expressing neurons to mood regulation (Zhong et al., 2014; Fritz et al., 2015). Notably, DA-expressing neurons may play an important role in the affective changes in other conditions, including systemic inflammation. When inflammation is significant, such as during illness, individuals often report impaired cognitive function and depressed mood. Recently, DREADDs were used to explore the contribution of DA-expressing neurons to the depressed mood that is linked to systemic inflammation (Fritz et al., 2015). Depressed mood was modeled in animals using the conditioned place aversion task. When a particular location is paired with an unpleasant experience, such as systemic inflammation, that location becomes less preferred (more aversive) relative to other neutral locations. It was observed that the excitation of DA-expressing neurons in the ventral midbrain blocked the establishment of conditioned place aversion.

The neural mechanisms governing anxiety, or more specifically fear, have also been studied using DREADD techniques. Importantly, animals have the capacity for both innate fears (e.g., to predators) and learned fear (e.g., to stimuli associated with aversive experiences). In certain situations, animals are faced with only two options for action; one of these options may elicit innate fear whereas another may elicit learned fear. In these cases, animals must suppress either innate fear or learned fear in order to select the best course of action (they must choose between ‘the lesser of two evils’ in order to survive). The ability to prioritize one fear over the other fear is thought to be mediated by the amygdala of the brain. Recently, Isosaka et al. (2015) demonstrated that serotonin type 2A (5HT2A)-receptor expressing cells in the central amygdala are centrally involved in the hierarchal organization of fear responses. Inhibition of 5HT2A cells with hM4Di receptors results in the suppression of learned fear and the enhancement of innate fear.

### Pain

Recently, DREADDs have been used to investigate the brain and spinal circuits that underlie pain behaviors. Pain is a complex, subjective phenomenon that causes feelings of physical and emotional discomfort; its conditions are heterogeneous and the underlying aetiologies, mechanisms, and sites of symptoms are diverse. Within the last 50 years, few effective treatments for chronic pain have emerged, largely because the mechanisms of these disorders are incompletely understood. This lack of insight is astounding when we consider that chronic pain affects approximately 25% of the population (Elliott et al., 1999). Since pain is the most common reason for seeking medical attention, the clinical and translational significance of DREADDs has potential to greatly progress the field. Despite the promising implications of DREADD technology, there are only a limited number of studies that use DREADDs to dissect pain circuits (Bourane et al., 2015; Jurik et al., 2015; Peirs et al., 2015). However, with the ease of implementing DREADD technology in the laboratory, the pain field is likely to see a surge in the use of DREADDs. This development may ultimately lead to a better understanding of pain circuits and the identification of new therapeutic targets.

Recently, the laboratory of Rebecca Seal was able to demonstrate that specific microcircuits within lamina III of the dorsal horn of the spinal cord encode mechanical pain sensitivity differently depending on the injury (Peirs et al., 2015). Neurons in these circuits are required for mechanical pain sensitivity and transiently express the vesicular glutamate transporter, VGLUT3, which packages glutamate into synaptic vesicles. In order to determine whether VGLUT3-expressing dorsal horn neurons convey mechanical pain hypersensitivity, a DREADD approach was used wherein hM3Dq receptors were specifically expressed in lamina III of the dorsal horn of *Vglut3-Cre* mice. DREADD-induced activation of lamina III VGLUT3-containing neurons induced mechanical hypersensitivity and allodynia (but did not affect heat hypersensitivity), an effect that was absent in mice lacking VGLUT3. Further, the application of CNO to lamina III neurons prepared from spinal cord slices produced action potential firing and an inward current, verifying the specificity of hM3Dq receptors into lamina III dorsal horn neurons. This is a key finding because it indicates that activating VGLUT3 neurons in lamina III, a region important for touch but largely ignored for its role in pain, is sufficient to drive activity of the downstream circuit that promotes mechanical allodynia.

There are also spinal circuits within the dorsal horn that mediate chronic itch, and as such the mechanisms underlying pain and itch are closely related (Luo et al., 2015). The sensation of itch is normally suppressed by inputs from mechanoreceptors. In many forms of chronic itch, including alloknesis, this gating mechanism is lost. Mice in which dorsal horn neuropeptide Y (NPY)-derived interneurons are genetically ablated or silenced with DREADDs develop mechanical itch without an increase in sensitivity to chemical itch or pain (Bourane et al., 2015). Specifically, hM4Di:NPY-*Cre* mice show mechanical itch when treated with CNO. The itch phenotype dissipates approximately 6 h following CNO injection, which coincides with the pharmacokinetics of DREADDs.

In another study examining spinal pain circuits, hM4Di receptor expression was restricted to neurons expressing transient receptor potential cation channel subfamily V member 1 (TRPV1), an ion channel important for conveying information regarding heat and pain (Saloman et al., 2015). TRPV1-*Cre* mice were used to restrict the transfection of hM4Di receptors to TRPV1-expressing cells, which include all C-fibers that innervate the spinal cord. The administration of CNO to hM4Di-TRPV1 mice resulted in a significant thermal analgesic phenotype that returned to baseline by 5 h post injection. However, calcium imaging and patch clamp experiments showed that sensory neurons from hM4Di-TRPV1 mice exhibit decreased resting intracellular Ca^2+^ and increased capsaicin-evoked Ca^2+^ even in the absence of CNO (Saloman et al., 2015). This CNO-independent effect in neurons from hM4Di-TRPV1 mice highlights the importance of mechanistic controls in DREADD studies. In contrast, we are unaware of any major cases in which the light-activated proteins routinely used for optogenetic experiments alter cellular properties in absence of light. However, there are cases in which prolonged light stimulation changes neuronal function (Herman et al., 2014; Mahn et al., 2016). For instance, sustained light application on channelrhodopsin 2-expressing neurons does not inactivate cells, but silences them. Certain types of cells, most notably interneurons, are particularly susceptible to this effect, which is termed ‘depolarization block’ (Herman et al., 2014).

DREADDs have also been used to understand the specific neurons in the brain that control pain responding and output. Brain structures can modulate incoming sensory information through a process called descending modulation (i.e., top–down modulation of pain). Descending inhibition may explain why soldiers do not appear to be in pain after battlefield injury, but later report pain after being taken to a hospital. Descending modulatory pathways can also facilitate pain perception in certain cases (Fields, 2004). In particular, the periaqueductal gray (PAG) sends projections to the rostral ventral medulla (RVM), which transmits these projections via the spinal cord dorsolateral funiculus to the dorsal horn. Activating hM3Dq receptors in PAG neurons that contain the dopamine transporter (DAT) resulted in significant thermal analgesia, while inhibiting PAG neurons containing GABA did not produce any analgesia (Taylor et al., 2014). As activation of dopamine neurons in the PAG did not increase anxiety, these cells may represent a novel target for treating chronic pain.

Further, in a rodent model of pancreatitis, inhibition of the paraventricular thalamic nuclei (PVT) with h4MDi receptors resulted in less pancreatitis-related abdominal pain and facial expressions (Jurik et al., 2015). DREADD-mediated PVT inhibition also decreased mPFC activity and increased RVM activation, pointing toward neuronal engagement of the descending inhibitory pain pathway (Jurik et al., 2015). There have only a limited number of studies that use DREADD tools to investigate the neural circuitry of pain, but these studies have been elegant and detailed. These reports collectively highlight the central, yet complex role of nociceptive processes within the central nervous system, and may stimulate the development of novel, centrally acting analgesic drugs for persistent pain conditions.

## DREADDs in Animal Models of Human Disease

DREADD techniques may eventually have uses beyond the deconstruction of behavior in a laboratory setting. Ultimately, these tools have considerable potential for the treatment of psychiatric disorders in humans (Urban and Roth, 2015). Given the recent demonstration that DREADDs may be expressed and functional in non-human primates, the possibility that these receptors can be used to modify behavior in humans now seems more achievable. Given this therapeutic potential, it is perhaps not surprising that there has been significant interest in using DREADD techniques to investigate animal models of human disease. This section will review recent studies utilizing DREADD techniques to explore the basis of human diseases, particularly those studies focusing on schizophrenia, autism, and Alzheimer’s disease.

Several groups have investigated the neural mechanisms underlying the behavioral changes in preclinical models of schizophrenia (Nguyen et al., 2014; Parnaudeau et al., 2015; Koike et al., 2016). Schizophrenia is a mental disorder characterized by abnormal social behavior, delusions, auditory hallucinations, and cognitive deficits. Abnormalities in GABAergic transmission, particularly within the vHPC and mPFC, are thought to contribute to the pathogenesis of the disorder (Braun et al., 2007). To explore this possibility, Nguyen et al. (2014) studied the effects of transiently suppressing the activity of PV-GABA neurons in the vHPC on mouse behavior. The authors observed that inhibiting PV-GABA neurons lead to behavioral deficits frequently observed in animal models of schizophrenia, including reduced pre-pulse inhibition of the startle response and impaired spontaneous alternation. In contrast, DREADD inhibition of a more heterogeneous GABA neuron population, those neurons expressing glutamic acid decarboxylase 65 (GAD65), resulted in different behavioral effects. This study highlights the specific roles that inhibitory neuron subtypes might play in different symptoms of schizophrenia.

Other studies have showed that cognitive deficits in schizophrenia, including impaired attention and cognitive flexibility, may be recapitulated in healthy animals through inhibition of the mPFC or its afferent targets (Parnaudeau et al., 2015; Koike et al., 2016). In one recent study, it was shown that inhibition of the anterior cingulate cortex, a part of the mPFC, produced a sustained deficit in attention as measured by the 5-choice serial response task (Koike et al., 2016). Additionally, inhibition of the mediodorsal thalamus, which is connected with the mPFC, impaired cognitive flexibility. Though DREADD approaches have been very useful in dissecting the circuitry of schizophrenia, there has yet to be a conclusive demonstration of how DREADD-based therapies may treat the symptoms of this disorder.

DREADD techniques have also been employed to study autism spectrum disorder (ASD). Notably, ASD is characterized by impaired social interaction as well as impaired verbal and non-verbal communication. Recently, the gene CNTNAP2 has been implicated in ASD, and transgenic mutant mice lacking CNTNAP2 have been used to model the disorder. CNTNAP2 knockout mice exhibit impaired social behavior, a hallmark of ASD. This deficit in social behavior was ameliorated by treatment with oxytocin or the selective activation of oxytocin-expressing neurons in the paraventricular nucleus of the hypothalamus using DREADD techniques (Penagarikano et al., 2015).

Alzheimer’s disease has also been examined using chemogenetic techniques, though not specifically DREADDs (Orr et al., 2015). In a recent study, astrocytes were targeted with chemogenetics because clinical data indicated that patients with Alzheimer’s disease exhibit increased expression levels of Gs-coupled adenosine receptor A2A in these particular cells. Orr et al. (2015) examined the role of A2A-receptor expressing cells in learning and memory deficits, which are commonly observed in Alzheimer’s disease. The conditional removal of A2A receptors on astrocytes, via transgenic techniques, enhanced long-term memory in mice. Conversely, the activation of A2A-receptor expressing cells using chemogenetic techniques lead to impaired memory performance. These findings not only identify a contributing factor for behavioral impairment in Alzheimer’s disease, but also identify a potential therapeutic strategy. Further, these results stand out as remarkable as they are one of a few recent cases in which chemogenetic techniques have achieved therapeutic benefit by targeting non-neuronal cells (Jain et al., 2013; Scofield et al., 2015).

DREADD technology has also been applied to the study of Down Syndrome, a disorder that is associated with an increased risk for Alzheimer’s disease (Fortress et al., 2015). Notably, Down Syndrome shares several neuroanatomical features with Alzheimer’s disease (including microglial activation, β-amyloid plaques, and neurofibrillary tangles) and also includes memory impairments (Teipel and Hampel, 2006). Interestingly, previous studies have shown that the locus coeruleus (LC), the main source of noradrenergic inputs to the rest of the brain, may be involved in the pathogenesis of both Down Syndrome and Alzheimer’s disease (Phillips et al., 2016). Recently, one group investigated the effects of selectively activating the LC in a mouse model of Down Syndrome, the Ts65Dn mouse. Ts65Dn mice exhibit a number of behavioral deficits reminiscent of Down Syndrome, including impaired working memory and hyperactivity. In this model, hM3Dq receptors were expressed in neurons of the LC. Treatment with CNO prior to behavioral testing, which is expected to activate hM3Dq-LC neurons, improved novel object recognition and reduced hyperactivity in Ts65Dn mice (Fortress et al., 2015).

## Conclusion and Future Directions

**FIGURE 4:**
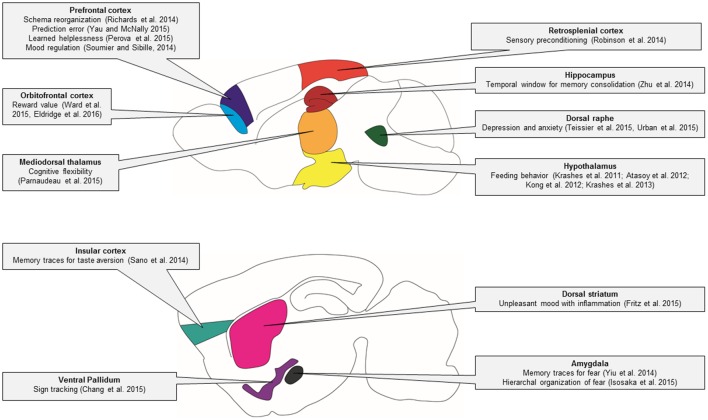
**Behavioral circuitry revealed by DREADD studies in mice.** In this schematic of the mouse brain, major brain regions that have been studied using DREADD technology are illustrated. The specific behavioral functions of these brain regions that have been identified using DREADD technology are indicated in the linked text.

Genetic techniques such as DREADDs and optogenetics are transforming many areas of neuroscience. In this review, we have discussed practical applications and highlighted a wide array of studies that have used DREADDs to deconstruct the neural circuitry of behavior (summarized in **Figure 4**) and unravel the complex mechanisms of disease.

Using DREADDs to determine the contribution of specific cell types to pathological states may allow for the development of more rational – and effective – therapies. Ultimately, whether a DREADD therapeutic approach is appropriate depends upon the pathology under study. For instance, chronic pain may be well-suited to treatment through DREADD technology. CNO is an attractive alternative to current analgesics as it is an orally available drug, which may silence pain circuits, and thereby provide pain relief, for prolonged periods. Interestingly, optogenetics is already being explored as a potential treatment for pain and other conditions. Two start-up companies (Circuit Therapeutics and RetroSense Therapeutics) have plans to begin human trials using optogenetics to treat chronic pain and genetic blindness, respectively. Early reports indicate that RetroSense Therapeutics have successfully treated their first patient. However, such therapies are in their early stages and it will take considerable refinement before they can be declared a success. Importantly, optogenetics requires the implantation of rigid light-emitting devices that could damage delicate nerve tissue. DREADD-based therapeutic approaches may be more feasible as such treatments only require the oral administration of CNO (or another synthetic drug). Before any DREADD-based therapies become a reality, however, we must first overcome the significant challenge of virally expressing designer receptors in adult human neurons. Only once this barrier has been broken can the full potential of DREADD-based therapeutic approaches be explored.

## Author Contributions

PW and LM conceived of the manuscript. PW and LM made the figures. All authors co-wrote and edited the manuscript.

## Conflict of Interest Statement

The authors declare that the research was conducted in the absence of any commercial or financial relationships that could be construed as a potential conflict of interest.The handling Editor declared a shared affiliation, though no other collaboration, with one of the authors LM and states that the process nevertheless met the standards of a fair and objective review.
